# Comparison of transbronchial needle aspiration with and without ultrasound guidance for diagnosing benign lymph node adenopathy

**DOI:** 10.1186/s13000-020-00958-4

**Published:** 2020-04-15

**Authors:** Hui Shen, Lingyun Lou, Ting Chen, Yi Zou, Bin Wang, Zhihao Xu, Qin Ye, Huahao Shen, Wen Li, Yang Xia

**Affiliations:** 1 Department of Respiratory and Critical Care Medicine, Second Affiliated Hospital of Zhejiang University School of Medicine, Hangzhou, Zhejiang 310009 China; 2 Department of Respiratory and Critical Care Medicine, Huzhou Central Hospital, Huzhou, Zhejiang 313000 China; 3 Department of Respiratory and Critical Care Medicine, Fouth Affiliated Hospital of Zhejiang University School of Medicine, Yiwu, Zhejiang 322000 China; 4 Department of Endoscopic Center, Second Affiliated Hospital of Zhejiang University School of Medicine, Hangzhou, Zhejiang 310009 China; 5 Department of Pathology, Second Affiliated Hospital of Zhejiang University School of Medicine, Hangzhou, Zhejiang 310009 China

**Keywords:** Transbronchial needle aspiration (TBNA), Endobronchial ultrasound TBNA (EBUS-TBNA), Transbronchial forceps biopsy (TBFB), Benign mediastinal lymphadenopathy

## Abstract

**Background:**

Transbronchial needle aspiration (TBNA) is a minimally invasive procedure performed to diagnose lymph node (LN) adenopathy. TBNA with and without endobronchial ultrasound (EBUS) guidance has a high diagnostic yield for malignant LN enlargement, but the value for diagnosing benign LN enlargement has been less thoroughly investigated.

**Methods:**

We retrospectively evaluated 3540 patients with mediastinal LN enlargement who received TBNA. One hundred sixty-six patients with benign mediastinal lymphadenopathy were included and 293 LNs were biopsied. A positive result was defined as a specific histological abnormality. Conventional TBNA (cTBNA) and EBUS-TBNA, as well as cTBNA and transbronchial forceps biopsy (TBFB), were compared. The subgroup analysis was stratified by disease type and LN size.

**Results:**

A diagnosis was made in 76.84% of the EBUS-TBNA and 61.31% of the cTBNA (*P* < 0.05). EBUS-TBNA was superior to cTBNA for both granulomatous (65.18% vs. 45.45%, *P* < 0.05) and non-granulomatous disease (96.92% vs. 84.06%, *P* < 0.05). In contrast, the diagnostic yield of EBUS-TBNA was higher than that of cTBNA for LNs < 20 mm (79.44% vs. 64.29%, *P* < 0.05), but for LNs > 20 mm the difference was marginal. These findings were confirmed in a group of independent patients who received cTBNA plus EBUS-TBNA. The diagnostic yield did not differ between cTBNA and TBFB, but significantly increased to 76.67% when both modalities were employed.

**Conclusions:**

EBUS-TBNA is the preferred minimally invasive diagnostic method for benign mediastinal LN disease. Combined cTBNA and TBFB is a safe and feasible alternative when EBUS is unavailable.

## Background

Transbronchial needle aspiration (TBNA) is a minimally invasive diagnostic modality for patients with mediastinal and hilar diseases [1]. Endobronchial ultrasound (EBUS) allows for real-time localization and aspiration of lymph nodes (LNs) during bronchoscopy, which improves the safety and accuracy of TBNA. Both conventional TBNA (cTBNA) and EBUS-guided TBNA (EBUS-TBNA) are associated with a high diagnostic yield for malignant mediastinal LN enlargement [2–5]. However, the diagnostic value of the two modalities for benign mediastinal lymphadenopathy has been less thoroughly investigated. The diagnostic efficiency of the various biopsy methods remains controversial. The reported diagnostic rate of cTBNA in patients with benign mediastinal LN enlargement varies between 21.4 and 76% [6–11], while that of EBUS-TBNA varies between 74.5 and 96% [12–19]. However, these studies used single-arm designs and had relatively small sample sizes. The main determinants of the diagnostic rate for cTBNA are the operative technique and the type of specimen (tissue or cytology) obtained by needle aspiration. Although EBUS improves the diagnostic rate of cTBNA, puncture specimens are usually a mixture of cell masses and blood clots that are not always morphologically representative. A few studies have introduced the technique of transbronchial forceps biopsy (TBFB) as a potential option to obtain a large volume of biopsied tissue, which could aid in the diagnosis of benign mediastinal lymphadenopathy [20–23]. However, the sample size of these studies was somewhat limited and the reported diagnostic rates varied greatly. Most studies were conducted under EBUS guidance and the diagnostic efficiency of TBFB without EBUS remains unknown. More importantly, no study has directly compared TBFB and TBNA. Thus, the role of TBNA, including cTBNA and EBUS-TBNA, in the diagnosis of benign thoracic LN enlargement is not known. Whether TBFB is superior to TBNA for evaluating mediastinal and hila lymphadenopathy remains to be determined. We retrospectively analyzed 3540 patients with benign mediastinal lymphadenopathy. Systematic comparison of cTBNA and EBUS-TBNA, as well as of cTBNA and TBFB, was carried out. We aimed to determine the diagnostic role of TBNA and TBFB in patients with benign mediastinal lymphadenopathy.

## Material and methods

### Patient selection

**Fig. 1:**
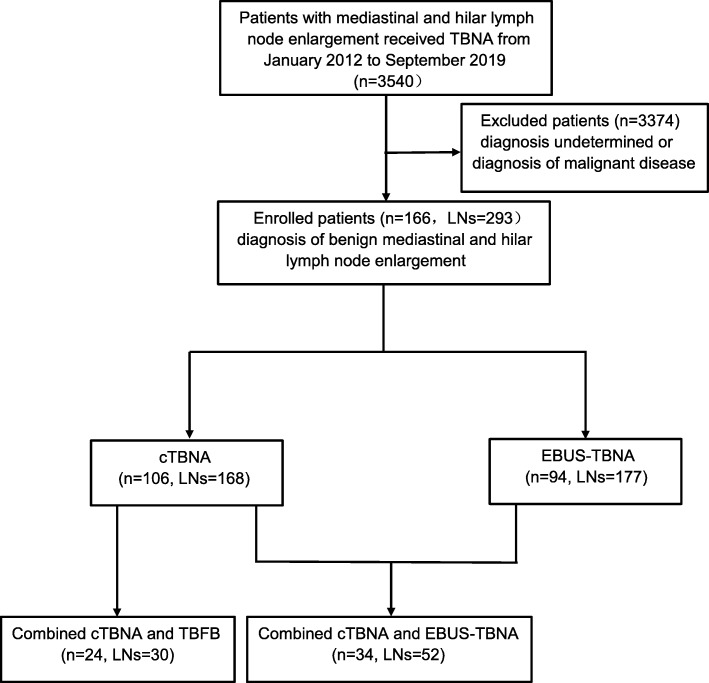
Flow diagram of patient inclusion

We retrospectively reviewed 3540 patients with mediastinal and hilar LN enlargement who received TBNA, registered in the inpatient system of the Second Affiliated Hospital of Zhejiang University School of Medicine from January 2012 to June 2019. Based on a careful review of the patients’ medical records (medical history, laboratory results, chest computed tomography (CT) scans, tracheoscopy, and cytological and histological results), 166 patients diagnosed with benign mediastinal LN enlargement who underwent cTBNA or EUBS-TBNA were included in the study. Patients were excluded if there was no final diagnosis or the pathology report noted a malignancy (Fig. 1). The size of the LNs was measured on CT or EBUS images.

### Performance of conventional Transbronchial needle aspiration

We evaluated the patients and localized the punctured LN based on the chest CT and Wang’s map [24]. Experienced bronchoscopists performed cTBNA under local or general anesthesia in 168 LNs. Once the LN had been located, a 19-gauge TBNA needle (MW-319; Conmed, Utica, NY, USA) was inserted through the bronchial wall. Negative pressure was applied, and the aspiration biopsy specimen was collected. The needle was quickly moved back and forth, and each LN was punctured three times.

### Performance of Endobronchial ultrasound-guided trans-bronchial needle aspiration

The EBUS-TBNA examination was conducted after routine bronchoscopy in 177 LNs using an EBUS-TBNA bronchoscope (BF-UC 260FW; Olympus, Tokyo, Japan). The LNs were detected and measured using an ultrasound scanner (EU-ME1; Olympus). A 22-gauge needle (NA-201SX-4022; Olympus) was employed for the LN biopsy under real-time visualization. Each LN was biopsied three times.

### Performance of Transbronchial forceps biopsy

TBFB plus TBNA was performed in 30 LNs from 24 patients. cTBNA was carried out first, and a 21-gauge biopsy forceps (M00515180; Boston Scientific, Cambridge, MA, USA) was then passed through the opening in the bronchial wall. The forceps were opened and pushed forward slightly, and then closed to finish the biopsy. Each LN was biopsied twice.

### Pathological analysis

Each aspirate obtained by TBNA or TBFB was either smeared onto numbered glass slides or placed into PreservCyt solution (ThinPrep; Hologic Inc., Marlborough, MA, USA). Then we picked out the visible tissue like materials from the smeared slides and PreservCyt solution, and the specimens were fixed in 4.0% buffered formalin solution and delivered to the pathology department, where they underwent dehydration, paraffin embedding, and sectioning. Meanwhile, we sent the smeared slides and rest of PreservCyt solution to the cytology laboratory for cytological analysis. Two experienced pathologists evaluated the cytological and histological results independently after hematoxylin and eosin staining using standard methods. Immunohistochemistry was performed where required for definitive diagnosis.

### Statistical analysis

The baseline characteristics of all enrolled patients are provided in Table 1. The diagnostic yield was calculated as a percentage. The diagnostic yields of cTBNA and EBUS-TBNA were compared using the chi-square test for independent samples, as were those of TBFB and cTBNA. In addition, univariate and multivariate logistic regression analyses were performed to identity factors affecting the diagnostic yield of cTBNA and EUBS-TBNA, respectively. A univariate logistic regression analysis of TBNA was also performed. SPSS software (version 20.0; SPSS Inc., Chicago, IL, USA) was used for the statistical analysis. A *P*-value < 0.05 was considered significant.

**Table 1:** Baseline Characteristics of Patients and Final Diagnosis

Total patients	166
Age, mean y[95%CI]	54 (52.06–55.93)
Male gender, No. (%)	76 (45.78%)
Smoking history, No. (%)	
Current smoking	25 (15.06%)
Former smoking	25 (15.06%)
Never smoking	116 (69.88%)
Lymph node size	
< 20 mm	173
≥ 20 mm	120
Diagnosis	
Granulomatous diseases	94
Sarcoidosis	83
Tuberculosis	10
Churg-strauss syndrome	1
Non-granulomatous diseases	72
Reactive lymphocytosis	60
Silicosis	8
Others^a^	4

^a^ Others include castleman disease (*n* = 1), paragonimiasis (*n* = 1), pneumomycosis (*n* = 2)

## Results

### Patient characteristics

A total of 166 patients (76 men and 90 women) with a definitive diagnosis of benign hilum of the lung or mediastinal lymphadenectasis were included in the final analysis. The mean age of the patients was 54 years (95% confidence interval [CI] 52.06–55.93). The mean LN size was 2.05 cm (95% CI 1.97–2.14). There were 25 current (21.6%), 25 former (21.6%), and 116 never smokers (56.9%). The 166 benign cases were divided into 94 with granulomatous disease and 72 with non-granulomatous disease. All patients underwent TBNA (either EBUS-TBNA or cTBNA, or both) and/or TBFB, and 293 LNs were biopsied. cTBNA and EBUS-TBNA were performed in 106 patients with 168 LNs and 94 patients with 177 LNs, respectively. Prior cTBNA followed by EBUS-TBNA was carried out in 34 patients with 52 LNs, and both cTBNA and TBFB were conducted in 24 patients with 30 LNs. The detailed demographic data are shown in Table 1.

### Diagnostic yields of TBNA

**Fig. 2:**
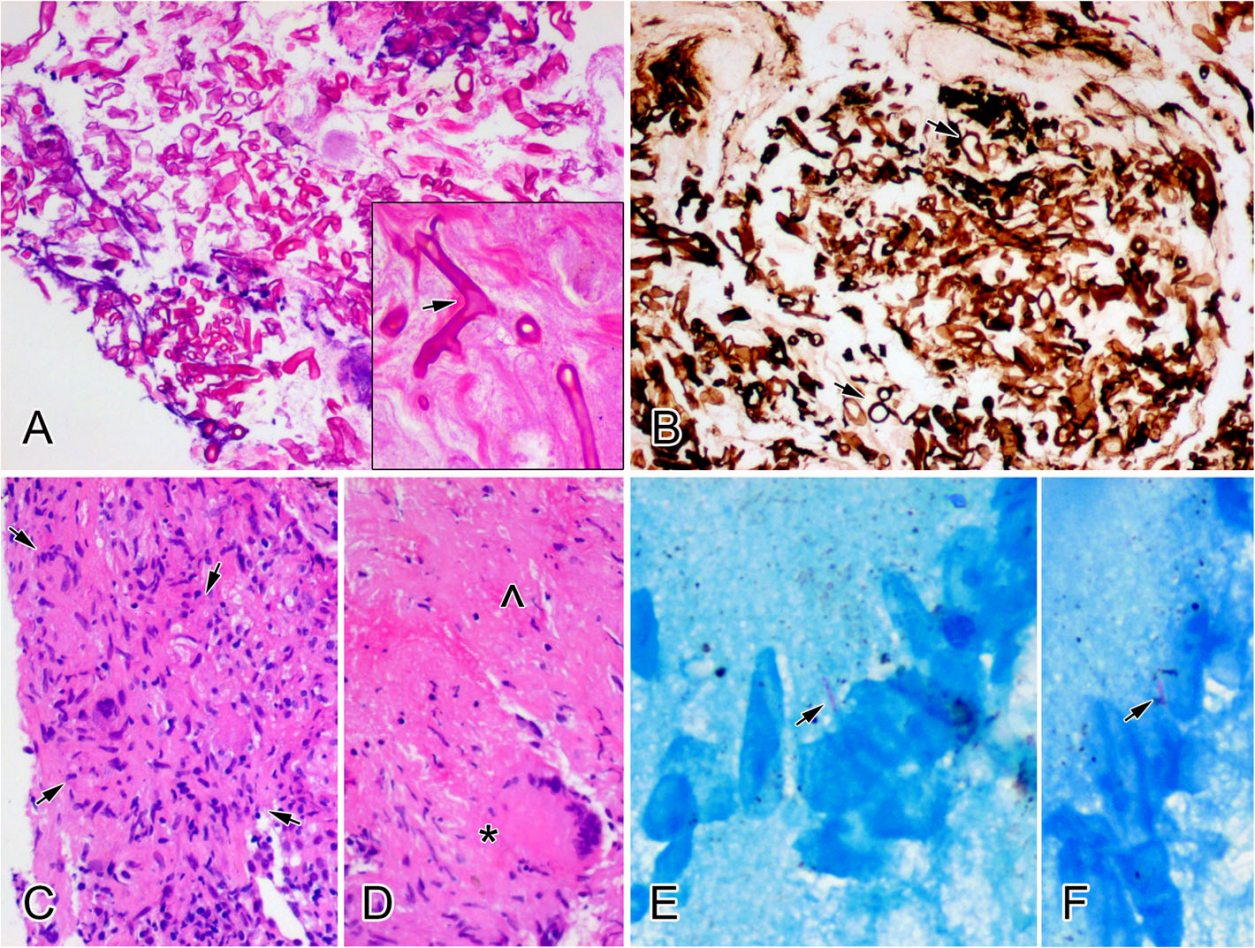
Morphological manifestations of two representative cases with lymph node mucormycosis and tuberculosis, respectively. Broad, non-septate and thick-walled hyphae are seen with right angle branching (arrow), indicating mucormycosis (**a**. H&E staining × 200). Cyst-like sections (arrows) of the Mucor hyphae are more prominent on staining of periodic acid-silver methenamine (PASM) (**b**. PASM staining × 200). Aggregation with epithelioid cells (inside arrows,) is present (**c**. H&E staining × 400), accompanied with typical Langerhans giant cells (*) and caseous necrosis (^), indicating tuberculosis (**d**. H&E staining × 400). Positive bacilli (arrows) of acid-fast staining are found (**e**, **f**. acid-fast staining × 400)

In this retrospective study, the overall diagnostic yield was 73.37% (215/293). The diagnostic rate of EBUS-TBNA for hilum of the lung and mediastinal adenopathy was 76.84% (136/177), while that of cTBNA was 61.31% (103/168) (Table 2). For example, pathology analysis showed aggregation of epithelioid cells accompanied with typical Langerhans giant cells and caseous necrosis in conjunction with positive bacilli of acid-fast staining, indicating the diagnosis of tuberculosis (Fig. 2). For cytological evaluation, 69.28%(115/166)cases used thinprep processing and 30.72% (51/166)cases used direct smears. Cytology demonstrated epithelioid cell formation was defined as positive for cases with granulomatous disease. The overall diagnostic yield of cytologic examination for granulomatous disease was 21.28%(20/94). More specifically, 12%(3/25)and 24.62%(17/69)for direct smears and thinprep treated slides, respectively. These data suggest that TBNA is an effective diagnostic modality for benign intrathoracic LNs and cytopathology serves as a reliable tool for diagnosis evaluation.

**Table 2:** Overall Diagnostic Rate of cTBNA and EBUS-TBNA

	cTBNA (*n* = 168)	EBUS-TBNA(*n* = 177)	*P* value
Overall diagnostic rate	103/168(61.31%)	136/177(76.84%)	0.002
Diagnosis			
Granulomatous	45/99(45.45%)	73/112(65.18%)	0.004
Non-granulomatous	58/69(84.06%)	63/65(96.92%)	0.003
Size			
< 20 mm	63/98(64.29%)	85/107(79.44%)	0.016
≥ 20 mm	40/70(57.14%)	51/70(72.86%)	0.051

n is the number of lymph nodes; *cTBNA* Conventional transbronchial needle aspiration; *EBUS-TBNA* Endobronchial ultrasound-guided transbronchial needle aspiration

### Comparison of EBUS-TBNA and cTBNA

We compared the diagnostic efficiency of cTBNA and EBUS-TBNA for benign LN adenopathy. As shown in Table 2, EBUS-TBNA had a significantly higher diagnostic yield than cTBNA (76.84% vs. 61.31%, *P* < 0.05).

We also investigated the factors potentially driving the difference in diagnostic efficiency between the modalities. The overall diagnostic yield of TBNA was significantly higher in non-granulomatous cases than granulomatous cases. More specifically, the diagnostic yield derived for both EBUS-TBNA and cTBNA was greater in patients with non-granulomatous inflammation. Of note, the diagnostic rate of EBUS-TBNA for granulomatous inflammation was higher than that of cTBNA (65.18% vs. 45.45%, *P* = 0.004). Similarly, EBUS-TBNA also had a higher diagnostic rate for non-granulomatous inflammation than cTBNA (96.92% vs. 84.06%, *P* = 0.003) (Table 2). We also stratified the LNs by size; 293 LNs were grouped according to a cut-off value of 20 mm. The diagnostic rate of EBUS-TBNA was 79.44% (85/107) for LNs > 20 mm and 72.86% (51/70) for LNs < 20 mm, while the respective rates for cTBNA were 64.29% (63/98) and 57.14% (40/70). The superiority of EBUS-TBNA over cTBNA was significant for LNs < 20 mm in size (*P* < 0.05), but it was only marginally superior for LNs > 20 mm (*P* = 0.051) (Table 2). In the 52 LNs for which cTBNA was followed by EBUS-TBNA, EBUS-TBNA had a better diagnostic yield than cTBNA (78.85% vs. 48.08%, *P* < 0.05) (Table 3), including for LNs < 20 mm; the diagnostic yield was comparable for LNs > 20 mm (*P* > 0.05, Table 3). These data suggest that EBUS guidance significantly increases the yield of TBNA in benign LN adenopathy. However, both EBUS-TBNA and cTBNA remain as viable options for diagnosing LNs > 20 mm.

**Table 3:** Diagnostic Rate of cTBNA and EBUS-TBNA in the Paired Design Group

	cTBNA(*n* = 52)	EBUS-TBNA(*n* = 52)	*P* value
Diagnostic accuracy	25/52(48.08%)	41/52(78.85%)	0.001
Size			
< 20 mm	15/32(46.88%)	25/32(78.13%)	0.002
≥ 20 mm	10/20(50%)	16/20(80%)	0.07

n is the number of lymph nodes; *cTBNA* Conventional transbronchial needle aspiration; *EBUS-TBNA* Endobronchial ultrasound-guided transbronchial needle aspiration

Granulomatous inflammation was positively correlated with the TBNA diagnosis on univariate logistic regression analysis (*P* < 0.001, Table 4). Previous pulmonary disease and granulomatous inflammation were significantly associated with the EBUS-TBNA diagnosis on univariate logistic regression analysis, while age, gender, LN size, smoking history, white blood cell count, and C-reactive protein were unrelated to the EBUS-TBNA diagnosis (Table 4). Only granulomatous inflammation was positively correlated with the EBUS-TBNA diagnosis on multivariate logistic regression analysis (*P* < 0.001, Table 4); it was also significantly associated with the diagnostic rate of cTBNA on univariate logistic regression analysis (*P* < 0.001, Table 4).

**Table 4:** Logistic regression analysis of TBNA, cTBNA and EBUS-TBNA

	TBNA		cTBNA		EBUS-TBNA			
Variables	Univariate OR (95%CI)	Univariate *P* value	Univariate OR (95%CI)	Univariate *P* value	Univariate OR (95%CI)	Univariate *P* value	Multivariate OR (95%CI)	Multivariate *P* value
Age	0.993 (0.973 ~ 1.014)	0.534	1.007 (0.983~1.031)	0.588	0.987 (0.957~1.017)	0.393	–	–
Leukocyte (10^9/L)	0.995 (0.884~1.12)	0.929	0.947 (0.814~1.102)	0.48	1.112 (0.934~1.323)	0.234	–	–
N%	1.016 (0.993~1.039)	0.174	1 (0.973~1.028)	0.991	1.025 (0.995~1.057)	0.106	–	–
E%	0.944 (0.875~1.018)	0.137	0.912 (0.812~1.024)	0.118	0.964 (0.875~1.063)	0.467	–	–
CEA (ng/ml)	0.927 (0.785~1.095)	0.373	0.904 (0.757~1.079)	0.264	1.082 (0.811~1.444)	0.592	–	–
CA125 (U/mL)	1.003 (0.995~1.01)	0.48	1.002 (0.992~1.011)	0.747	1.001 (0.993~1.008)	0.878	–	–
CA199 (U/mL)	0.993 (0.976~1.01)	0.396	0.987 (0.963~1.01)	0.269	1.007 (0.976~1.039)	0.655	–	–
SCC (ng/ml)	0.909 (0.622~1.328)	0.622	1.036 (0.69~1.557)	0.863	1.159 (0.428~3.135)	0.772	–	–
CRP (mg/L)	1.002 (0.994~1.011)	0.591	1 (0.99~1.011)	0.946	1.002 (0.991~1.014)	0.69	–	–
Lymph node size (cm)	0.856 (0.615~1.19)	0.354	0.937 (0.635~1.382)	0.743	0.832 (0.509~1.359)	0.462	–	–
Male	1.392 (0.418~4.634)	0.59	0.854 (0.458~1.593)	0.619	0.943 (0.461~1.926)	0.871	–	–
Smoking history	0.8 (0.252~2.537)	0.705	0.751 (0.376~1.499)	0.417	1.719 (0.73~4.045)	0.215	–	–
History of lung disease	2.207 (0.891~5.462)	0.087	1.062 (0.36~3.137)	0.913	0.223 (0.064~0.773)	0.018	0.263 (0.067~1.031)	0.055
Granulomatous inflammation	5.923 (2.967~11.826)	< 0.001	6.327 (2.97~13.479)	< 0.001	16.829 (3.907~72.496)	< 0.001	16.185 (3.737 ~ 70.1)	< 0.001

*TBNA* Transbronchial needle aspiration; *cTBNA* Conventional transbronchial needle aspiration; *EBUS-TBNA* Endobronchial ultrasound-guided transbronchial needle aspiration

### Comparison of cTBNA, TBFB, and the combined approach

The above data show that cTBNA is less efficient for diagnosing benign lesions. Thus, we aimed to determine how to improve the diagnostic rate of cTBNA for benign lesions without EBUS guidance. Pilot data indicated that TBFB might be useful for revealing enlargement of benign mediastinal LNs, and the diagnostic yield under EBUS guidance was even higher than that of EBUS-TBNA. In the pilot study, 24 patients (30 hila or mediastinal lymphadenectasis) received both cTBNA and TBFB. The diagnostic rate was 63.33% (19/30) using cTBNA alone, 53.33% (16/30) using TBFB alone, and 76.67% (23/30) using cTBNA plus TBFB (Table 5). Of note, no severe complications occurred as a direct result of TBFB. Among these patients, there was an interesting case who was negative for TBNA, but the specimen biopsied via TBFB exhibited broad, non-septate and thick-walled hyphae with right angle branching, indicating mucormycosis (Fig. 2). Our data suggest that TBFB is a safe and feasible option for diagnosing enlargement of benign LNs, and cTBNA alone was not notably superior. cTBNA combined with TBFB is a potential option when EBUS is not available.

**Table 5:** Diagnostic rate of cTBNA and TBFB in the paired design group

	cTBNA(*n* = 30)	TBFB(*n* = 30)	Combined (*n* = 30)	*P* value ^a^	*P* value ^b^
Diagnostic accuracy	19/30(63.33%)	16/30(53.33%)	23/30 (76.67%)	0.549	0.016

^a^ cTBNA vs. TBFB ^b^ TBFB vs. combined cTBNA+TBFB

n is the number of lymph nodes; *cTBNA* Conventional transbronchial needle aspiration; *TBFB* Transbronchial forceps biopsy

## Discussion

The current study provides strong evidence of the effectiveness of cTBNA, EBUS-TBNA, and TBFB for diagnosing benign mediastinal lymphadenopathy. The major findings of this retrospective comparative study were as follows: TBNA was effective for revealing enlargement of benign LNs; EBUS-TBNA had a higher diagnostic yield than cTBNA, but cTBNA was not inferior for LNs > 20 mm; and TBFB without EBUS guidance is a safe and feasible option for diagnosing benign LN adenopathy. However, combined cTBNA and TBFB achieved a higher diagnostic yield. Our study provides solid evidence that EBUS-TBNA is the optimal approach for diagnosing benign LN enlargement, although combined cTBNA and TBFB is a viable alternative when EBUS guidance is not available.

Both cTBNA and EBUS-TBNA are important tools for diagnosing mediastinal and hilar lymphadenopathy, including benign and malignant diseases [25]. A large number of prospective trials have demonstrated that EBUS-TBNA, as the gold standard, is similar to surgical mediastinoscopy in sensitivity and pathological staging [26–28]. EBUS-TBNA had a sensitivity of 89% for lung cancer and was recommended by the guidelines to stage the mediastinum [29]. Likewise, for peripheral pulmonary nodules, ultrasonography (US) also plays a critical role. US-guided transthoracic fine-needle aspiration demonstrated elevated clinical applicability and diagnostic utility compared to CT-guided transthoracic fine-needle aspiration [30]. Moreover, the materials obtained by needle aspiration can be further evaluated for molecular pathological diagnosis through various approaches, such as next generation sequencing, multiplex fluorescence in situ hybridization and so on [31–33]. However, there have been few studies on TBNA for patients with benign lymphadenopathy. TBNA has been shown to be useful for diagnosing benign lymphadenopathy, with diagnostic rates of 51.6–96% [12–19, 23, 34–36], but these studies had obvious limitations. First, most used a single-arm design, reporting only the diagnostic rate of EBUS-TBNA for benign disease, without any comparison to cTBNA [13, 14, 17–19, 23, 34–36]. Second, the sample size of some of the studies was small; further studies with larger samples are needed size to confirm the diagnostic accuracy of EBUS-TBNA [13, 15, 16, 18, 19, 23, 34, 35]. Third, most studies enrolled both malignant and benign lymphadenectasis patients, rather than specifically focusing on benign lymphadenopathy [13, 17, 19, 23, 36]. In addition, studies performed in western countries were mainly concerned with sarcoidosis, whereas those done in Asian countries focused on tuberculosis [12, 14–16, 18, 34, 35]. In our study, we tried to address the shortcomings of the previous investigations. We enrolled patients with various kinds of benign disease, and also performed a subgroup analysis to determine the indications for cTBNA in individuals with suspected benign LN adenopathy. In our study, the diagnostic yield was 73.37% for TBNA; more specifically, it was 61.31% for cTBNA and 76.84% for EBUS-TBNA. Although EBUS-TBNA was superior, cTBNA also showed good diagnostic power, and was not for LNs > 20 mm. In the univariate and multivariate logistic regression analyses, only granulomatous inflammation was significantly associated with the diagnostic efficiency of TBNA. Based on both previous studies and our findings, EBUS-TBNA should be considered as the first-line modality for diagnosing enlargement of benign mediastinal LNs. cTBNA remains a valuable technique with acceptable (> 60%) sensitivity, particularly for larger LN lesions.

It is worth noting that EBUS-TBNA requires specialized, relatively expensive equipment, and thus is not available in many hospitals, particularly those in developing countries. Hence, it is important to explore ways to achieve higher diagnostic efficiency without EBUS. We aimed to devise a safe, efficient, feasible and easy-to-perform method to meet this need. In this study, we confirmed the diagnostic value of cTBNA; the poorer performance of cTBNA in the granulomatous cases was mainly due to the small volume of specimens available to support the histological findings. Forceps biopsy is a classic clinical approach used to obtain larger-volume specimens than needle biopsies. Several pilot studies reported a diagnostic yield of EBUS-guided mediastinal LN forceps biopsy above 80% for nonmalignant disease [20–23]. However, in our study the diagnostic yield of TBFB without EBUS for benign disease was only 53.33% (16/30). No significant difference in yield was noted between cTBNA and TBFB. The low diagnostic rate of TBFB could have occurred for several reasons. First, the tip of the biopsy forceps is too blunt to effectively penetrate the targeted LNs; thus, a positive TBNA result but negative TBFB (fibrofatty tissue) may be seen. Second, whether the forceps cups opened successfully following forceps insertion into the LNs could not be confirmed. TBFB requires a new “forceps-needle” integrated tool to overcome its shortcomings. Of note, we found that cTBNA and TBFB combined had a high diagnostic rate, even without EBUS. The two modalities are complimentary and their combined use does not result in additional damage or cost based on currently available tools. Further validation of the efficiency and safety of this protocol is needed.

In addition to sampling techniques, preparation technique is also a pronounced determinant for diagnostic performance. Accumulated evidence shows that conventional cytological evaluation is capable to provide diagnostic clues in non-granulomatous diseases, such as amyloidosis, necrotizing lymphadenitis [37, 38]. On the other hand, cytology is a more potent diagnostic tool for granulomatous disease. However, the efficacy varied, ranging from ~ 10 to 80% [6, 39]. Notably, our cytological efficacy is close to the lower range of reported results. It could be partially explained by our protocol that we pick out all tissue fragment for histology evaluation which deprive off much indicating information for cytologists. Additionally, it is noteworthy to highlight that cell block is an essential complement to conventional smear in providing a reliable diagnosis especially in resource limited settings, presenting dramatic advantages in histomorphology analysis.

Our study had several limitations. First, used a retrospective design and various confounders may have affected the results. Second, we performed EBUS-TBNA with a 21-gauge TBNA needle only, rather than a 19-gauge histology needle, so could not address the potential effect of needle size on sensitivity. Third, the number of patients with benign lymphadenopathy enrolled to determine the efficacy of TBFB was relatively small. The type of forceps used for TBFB is also worthy of more in-depth investigation. Several factors, such as stiffness, size of the forceps cup, design of the forceps tip, and even the putative forceps-needle integrated design, should be considered in future studies.

## Conclusions

Both cTBNA and EBUS-TBNA are feasible and safe, and can provide histological specimens of enlarged mediastinal or hilar LNs. Due to its high sensitivity, EBUS-TBNA is the preferred option for diagnosing enlargement of benign LNs. Combined cTBNA and TBFB is a viable alternative when EBUS is not available, due to its relatively high diagnostic rate. We strongly advocate the use of minimally invasive techniques for diagnosing of enlargement of benign mediastinal LNs.

## Data Availability

The datasets generated and analyzed during the current study are not publicly available due the institutional review board restricts the use of the datasets to the current study only.

## Abbreviations

TBNA: Transbronchial needle aspiration
cTBNA: Conventional transbronchial needle aspiration
EBUS-TBNA: Endobronchial ultrasound-guided transbronchial needle aspiration
TBFB: Transbronchial forceps biopsy
LNs: Lymph nodes
H&E: Hematoxylin and eosin
IHC: IMMUNOHISTOCHEMISTRY.
